# Molecular Imaging of Brown Adipose Tissue Mass

**DOI:** 10.3390/ijms22179436

**Published:** 2021-08-30

**Authors:** Jing Yang, Haili Zhang, Kadirya Parhat, Hui Xu, Mingshuang Li, Xiangyu Wang, Chongzhao Ran

**Affiliations:** 1 School of Engineering, China Pharmaceutical University, Nanjing 210009, China; 3220080845@stu.cpu.edu.cn (H.Z.); kadypr@163.com (K.P.); bob545788414@163.com (H.X.); limshuang@163.com (M.L.); l1063420710@163.com (X.W.); 2 Athinoula A. Martinos Center for Biomedical Imaging, Department of Radiology, Massachusetts General Hospital, Harvard Medical School, Room 2301, Building 149, Charlestown, Boston, MA 02129, USA

**Keywords:** brown adipose tissue, tissue mass quantification, activation-independent, rest state, molecular imaging, TSPO, metabolic disorders

## Abstract

Brown adipose tissue (BAT), a uniquely thermogenic tissue that plays an important role in metabolism and energy expenditure, has recently become a revived target in the fight against metabolic diseases, such as obesity, diabetes, and non-alcoholic fatty liver disease (NAFLD). Different from white adipose tissue (WAT), the brown adipocytes have distinctive features including multilocular lipid droplets, a large number of mitochondria, and a high expression of uncoupling protein-1 (UCP-1), as well as abundant capillarity. These histologic characteristics provide an opportunity to differentiate BAT from WAT using imaging modalities, such as PET/CT, SPECT/CT, MRI, NIRF and Ultrasound. However, most of the reported imaging methods were BAT activation dependent, and the imaging signals could be affected by many factors, including environmental temperatures and the states of the sympathetic nervous system. Accurate BAT mass detection methods that are independent of temperature and hormone levels have the capacity to track the development and changes of BAT throughout the lifetime of mammals, and such methods could be very useful for the investigation of potential BAT-related therapies. In this review, we focus on molecular imaging modalities that can detect and quantify BAT mass. In addition, their detection mechanism and limitations will be discussed as well.

## 1. Introduction

BAT is a specialized tissue for thermogenesis, and its function in mammals is to dissipate large amounts of chemical/food energy as heat, thus maintaining an energy balance of the whole body [1,2,3]. BAT had been considered to have no physiologic relevance in adult humans, even though it is highly abundant in embryonic and early postnatal stages. However, this dogmatic opinion has been overturned by large clinical studies. In 2009, Cypess et al. reported that, by analyzing 3640 PET/CT images of 1972 patients, BMI (body mass index) is inversely correlated with the amount of brown adipose tissue, strongly suggesting that BAT is an important target in obesity and diabetes [4]. Other important studies have also clearly endorsed the importance of BAT in adults [5,6,7,8,9,10,11,12,13,14,15,16], and the significance and potential benefits of BAT have strongly been supported by numerous groundbreaking studies since 2009 [17,18,19,20,21,22,23,24,25,26,27,28,29,30,31,32,33,34,35,36,37]. The most distinct characteristics of BAT include a large number of mitochondria, abundant uncoupling protein-1 (UCP-1) expression, numerous small oil droplets in a single cell, as well as significant vascularization of BAT tissue [1,38,39,40,41]. These characteristics strongly indicate that BAT plays an important role in metabolism and energy expenditure, and regulating BAT mass and activation represents a very attractive approach for obesity and diabetes treatment.

BAT mass is the actual weight of the tissue that is consistent with and without stimulations, such as cold exposure and beta-agonist treatment, while BAT activation, reflecting by imaging signals or functional measurement, is highly dependent on several factors, including temperature, hormones, diet, medications, and the states of the sympathetic nervous system [42,43,44,45,46,47,48]. Accurately reporting BAT mass is a tremendous challenge for imaging scientists, due to the fact that BAT mass and BAT activation are intertwined under various conditions [49,50]. It is obvious that there is no absolute “resting” status of BAT, and BAT activation cannot be “zero” for a living subject. Therefore, dissection of BAT mass and BAT activation is a remarkable challenge.

The standardized and reproducible non-invasive imaging methods for the assessment of BAT mass and activation can greatly benefit the monitoring of BAT-related therapeutic progress. BAT has a large number of mitochondria and the dense iron inside gives the tissue a brownish color. In contrast to white adipose tissue (WAT), BAT also contains numerous smaller oil droplets, a higher expression of UCP1, and more capillaries. These unique features have become useful biomarkers for differentiating BAT from WAT by using different imaging modalities. Most of the reported imaging modalities have the potential for quantitative imaging of BAT activation, while few of them could be used to report BAT mass that is activation independent. Therefore, the techniques which could quantify BAT mass independent of activation are highly desirable.

In this review, we performed an electronic search in PubMed and Web of Science with terms “brown adipose tissue imaging” and “brown adipose tissue mass imaging”. The searched results included studies in rodents and/or humans, and the reference lists of relevant articles were also surveyed, while the imaging techniques were listed and compared (Table 1). In this review, imaging techniques of positron emission tomography (PET), single photon emission computerized tomography (SPECT), magnetic resonance imaging (MRI), near-infrared fluorescence imaging (NIRFI), contrast enhanced ultrasound (CEUS), near-infrared spectroscopy (NIRS), and infrared thermography (IRT) were included. The methods that could provide imaging signals from BAT under thermoneutral conditions and the signal which was not influenced by varied temperature or drug treatment were classified as BAT mass imaging. Two types of techniques were defined as BAT activation imaging: (1) the techniques which could detect BAT at room temperature, but the signal was increased or decreased significantly under cold temperature or drug stimulation; (2) the techniques which could not detect BAT at thermoneutral condition unless stimulated by cold temperature or drug treatment. Although a number of prior reviews have discussed imaging of BAT [42,43,44,45,46,47], there is no specific review on BAT mass imaging. Therefore, in this systematic review, we have endeavored to identify the current imaging modalities that are independent of BAT activation state.

## 2. Imaging Modalities for BAT Detection

### 2.1. Positron Emission Tomography (PET)

PET is the most frequently used imaging method for the assessment of BAT. PET tracers targeting BAT were designed according to the unique characteristics of BAT, including dense mitochondria packing, high metabolic activity, and high expression of unique proteins, such as uncoupling protein-1 (UCP-1) and translocator protein (TSPO).

^18^F-FDG-PET/CT is currently the most frequently used and the best established method for visualizing activated BAT in humans [4,5,13,14]. However, ^18^F-FDG PET/CT is not able to detect BAT mass without stimulations, including cold temperature and drug treatment. Meanwhile, the amount of BAT that is detected by ^18^F-FDG-PET could be affected by many factors, including season/outdoor temperature, diet and medications. Therefore, the ^18^F-FDG-PET/CT experiments in humans have been standardized in BARCIST 1.0 criteria for the quantitative assessment of BAT [51].

PET tracers including ^18^F-THA [52,53], ^11^C-acetate [63], and ^15^O-O_2_ [66] that target BAT metabolism were also used to detect BAT activation, but not BAT mass. Their accumulations in BAT were increased after cold exposure. Interestingly, contrary to ^18^F-FDG PET imaging, the reported PET signal of ^18^F-FBnTP [55,56] was inversely correlated with BAT activation. ^18^F-F-DA, a dopamine analogue, was reportedly capable of visualizing BAT under a thermoneutral condition; however, whether its uptake in BAT would be affected by cold or drug stimulation was not investigated [54]. ^18^F-FMPEP-d2, that targets cannabinoid receptor-1, also has the ability to assess BAT deposits in a rest state, although the BAT metabolic state was not reported [57]. Among the reported PET tracers, only a few of them were able to detect BAT mass unaffected by activation. The PET tracers that could detect BAT mass independent of activation are discussed below.

**Figure 1:**
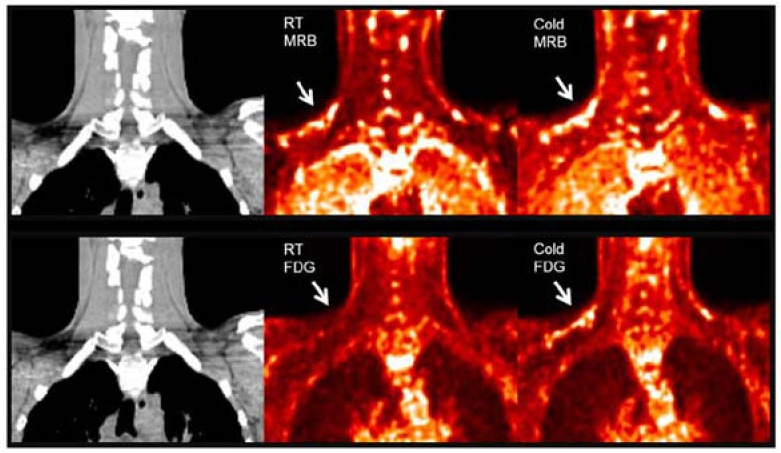
BAT imaging with ^11^C-MRB and ^18^F-FDG under room and cold temperature in male subject. ^18^F-FDG and ^11^C-MRB are scaled from SUV 0 (black) to SUV 2 (white). ^11^C-MRB images are computed from the average of frames acquired between 40 to 60 min post-injection. Original data with permission from reference [54].

**^11^C-MRB.** Norepinephrine is a major signal molecule regulating the metabolic activity in BAT. In 2012, Ding et al. performed a PET study in rats with ^11^C-MRB, which is a targeting norepinephrine transporter. Their results demonstrated that ^11^C-MRB could efficiently bind to BAT at thermoneutral conditions and the thermo-stimulating conditions had no significant effect on the tracer uptake, while ^18^F-FDG contrast could be observed only under cold conditions [64]. In 2015, they continued their study in humans with ten healthy Caucasian subjects [65]. Similar to the results of animal studies, the subjects showed equal BAT uptake under both room temperature and cold conditions (Figure 1). Their results indicated the possibility of using ^11^C-MRB for the monitoring of BAT mass in the development and treatment of metabolic disorders, such as obesity and diabetes. However, the human study has some limitations, including a small sample size, short half lifetime of ^11^C-labelled tracer, and low signal to noise ratio in human subjects. Additional studies are needed to determine its validity for BAT mass imaging both in rodents and human.

**Figure 2:**
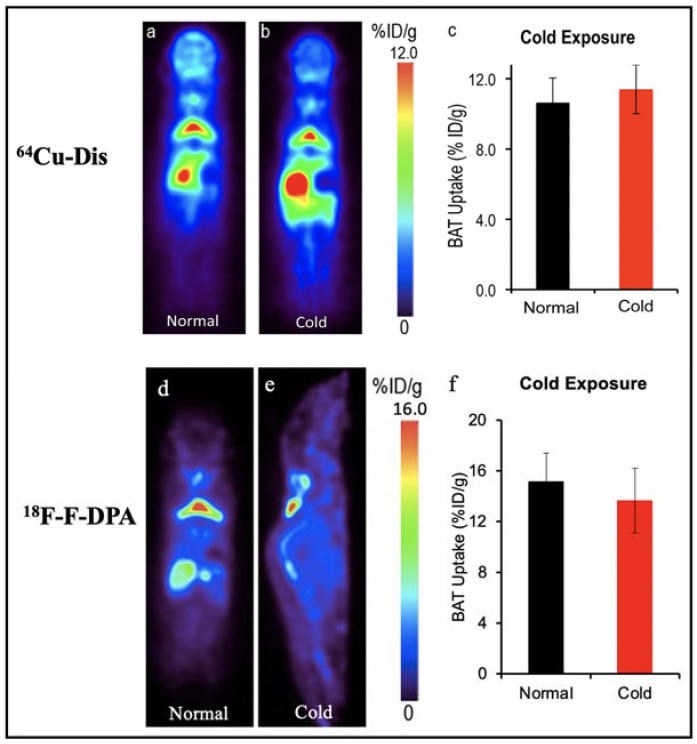
BAT imaging with TSPO PET tracer: ^64^Cu-Dis and ^18^F-F-DPA. (**a**) PET imaging with ^64^Cu-Dis in mouse under room temperature. (**b**) Representative coronal image under a cold exposure condition. (**c**) Quantitative analysis of (**a**,**b**). There was no significant difference in uptake between control and cold-treated groups (*p* = 0.359). (**d**) PET imaging with ^18^F-F-DPA in a mouse under room temperature. (**e**) Representative coronal image under a cold exposure condition. (**f**) Quantitative analysis of (**d**,**e**). There was no significant difference in uptake between control and cold treated groups (*p* = 0.356).

**^64^Cu-Dis, ^18^F-F-DPA and ^11^C-PBR28**. Our group have reported a synthesis-free PET imaging strategy for BAT mass [58]. After a top-down screening with a number of ^64^Copper ligands through a PET scanner, we found that the combination of ^64^CuCl_2_ with an FDA-approved drug for alcoholism, disulfiram, provided a considerable high contrast for BAT, and named the tracer as ^64^Cu-Dis. Importantly, the high BAT uptake of ^64^Cu-Dis was not affected by BAT activation (Figure 2). Interestingly, we found that TSPO, a transport protein located on the outer mitochondrial membrane, was the binding target of ^64^Cu-Dis. Although there is no published data regarding ^64^Cu-Dis use in humans, the high accumulation in BAT and the easy-to-use protocol (by only two step injection) suggested that ^64^Cu-Dis was a very promising PET tracer for quantitative BAT mass imaging.

Remarkably, our data also showed that ^18^F-F-DPA, a newly developed TSPO tracer, had considerable high uptake in BAT depots (~15% ID/g), which was consistent with ex vivo bio-distribution data [58]. Notably, there was no significant difference between cold treatment and those under thermo-neutral conditions, indicating that ^18^F-F-DPA could serve as a reliable tracer for BAT mass imaging regardless of its activation (Figure 2). To study the relationship of BAT and cancer cachexia, Huo et al. compared the ^18^F-FDG and ^18^F-F-DPA PET imaging of BAT with tumor-bearing mice [59]. Their data showed ^18^F-F-DPA have significant uptake in BAT without any stimulation, which was consistent with our previous report [58,62]. Moreover, they showed that the uptake of ^18^F-F-DPA by BAT in the late stage was significantly higher than that in the early stage.

**Figure 3:**
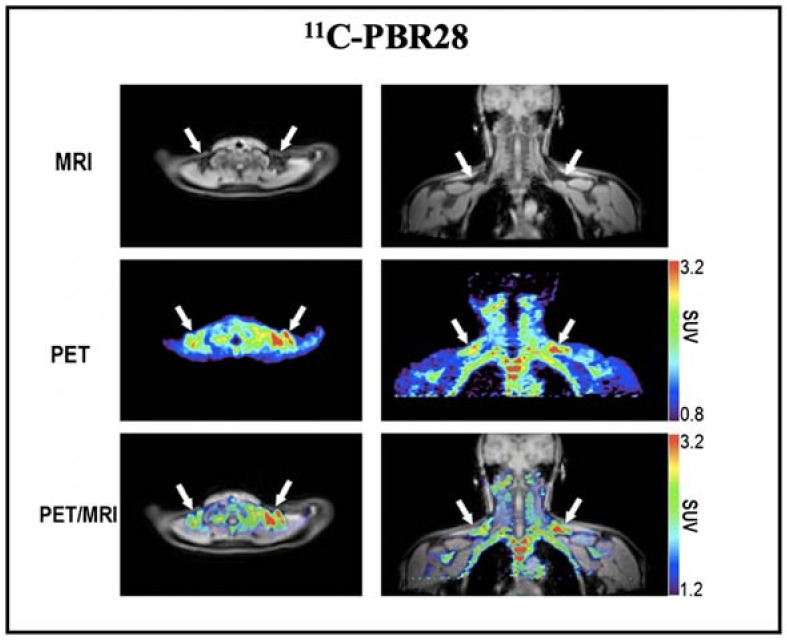
BAT imaging with TSPO PET tracer: ^11^C-PBR28. Representative images of a healthy volunteer who underwent PET/MRI using ^11^C-PBR28. The images were obtained 60–90 min after 14 m Ci of ^11^C-PBR28 injection. The supraclavicular BAT depots are indicated with arrows. Reproduced data with permission from reference [64].

Based on the above promising results, we also conducted a retrospective imaging analysis of PET/MRI images obtained after injection of ^11^C-PBR28 (a widely used TSPO tracer) in healthy volunteers [62]. The results showed high ^11^C-PBR28 accumulation in BAT depots under a thermoneutral condition (Figure 3). However, this study did not provide BAT imaging data under cold exposure, which is essential to assess whether the uptake of ^11^C-PBR28 was independent of activation. As Thompson et al. have shown, TSPO expression could not be altered by acute cold exposure [126], and we believe that TSPO has the great potential as a reliable biomarker for BAT mass imaging.

**Figure 4:**
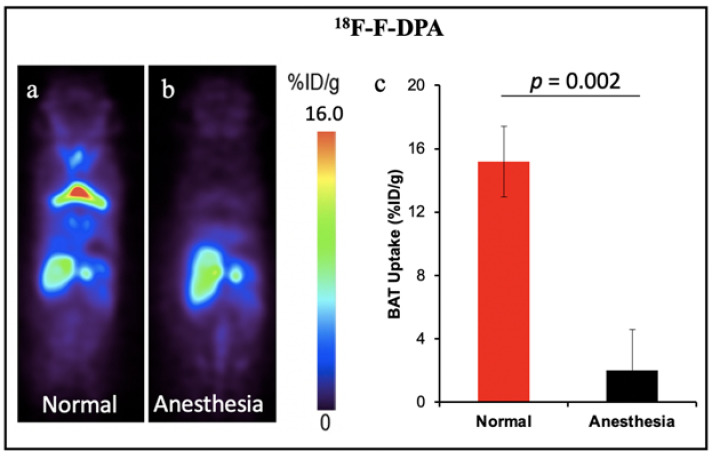
BAT imaging with TSPO PET tracer ^18^F-F-DPA: (**a**) PET imaging with ^18^F-F-DPA in mouse under room temperature. (**b**) Representative coronal image under light anesthesia for 30 min before tracer injection. (**c**) Quantitative analysis of (**a**,**b**). There was a significant difference in uptake between the control and anesthesia treated groups.

**Other TSPO PET tracers for BAT: ^18^F-FEPPA and ^18^F-PBR28.** In addition, other groups have demonstrated the feasibility of TSPO PET tracers for BAT imaging. Goggi et al. reported that ^18^F-FEPPA was able to detect inguinal WAT browning but not BAT activation in mice after dosing with β3-adrenergic agonist CL-316,243 [60]. Their results indicated ^18^F-FEPPA could be a promising tracer for BAT mass detection, independent of its metabolic state. Interestingly, Im et al. have demonstrated the ^18^F-PBR28 could detect BAT under thermoneutral conditions, however cold temperatures could increase the BAT uptake [61]. The results were contrary to our previous report which indicated the TSPO tracer could only detect BAT mass. Except for our retrospective study with ^11^C-PBR28 that was performed in humans, all the other TSPO tracers were tested in mice. The small number of mice could be one of the limitations for all the tests. Another reason causing the inconsistent results could be the anesthetic state of animals. Although the data from our group has not been published, during our PET imaging experiments we noticed that the isoflurane level and the duration of anesthesia had significant effects on the TSPO tracer uptake in BAT. High levels of isoflurane and long anesthesia duration could cause the “disappearance” of BAT in mice. As shown in Figure 4, 30 min anesthesia with isoflurane before tracer injection decreased the uptake of ^18^F-F-DPA in BAT by about eight-fold. In this regard, both the percentage of isoflurane and the anesthesia time should be kept consistent for each animal. Studies with a large number of animals and more studies in human with standardized conditions will benefit the investigation of BAT mass imaging with TSPO tracers in future research.

### 2.2. Single Photon Emission Computerized Tomography (SPECT)

Compared to PET imaging, SPECT radiotracers for BAT have longer half lifetimes and were usually used with higher radiation doses. Moreover, SPECT images have lower resolutions and quantification accuracy. Several SPECT studies on BAT imaging were retrospectively analyzed. Ishida et al. retrospectively analyzed 385 consecutive studies of ^99^mTc-tetrofosmin uptake in pediatric patients with cardiac disorders [67]. They found increased ^99^mTc-tetrofosmin uptake in the interscapular BAT in 17% of patients, and the uptake signal was significantly higher in winter than in spring or summer. Wahl et al. reviewed SPECT/CT scans using ^99^mTc-MIBI for parathyroid imaging in 205 patients [68]. Their study showed BAT uptake in 6.3% of patients, while the patients with ^99^mTc-MIBI uptake in BAT were younger than the patients with no ^99^mTc-MIBI uptake. Interestingly, other reported SPECT tracers all showed an increased uptake in BAT after cold exposure and drug treatment although they were able to detect BAT at room temperature [69,70], suggesting the current SPECT technique is not applicable for BAT mass imaging.

### 2.3. Magnetic Resonance Imaging (MRI)

Compared to PET and SPECT, MR imaging is a more attractive modality for human studies due to no ionizing-radiation. In recent years, developing MRI methods that can differentiate BAT and WAT has been an increasing interest [127,128,129,130]. The unique structure of BAT with multilocular oil droplets and dense mitochondria and abundant capillaries provides a unique basis for MRI to selectively image BAT via different mechanisms of generating image contrast. Reportedly, MRI is able to image the distribution, structure and function of BAT with different techniques. Among these techniques, chemical shift MRI such as fat fraction mapping and T2*-weighted mapping were able to measure BAT volume while Blood Oxygen Level Dependent (BOLD) MRI, hyperpolarized Xenon MRI, and contrast-enhanced MRI were employed to assess BAT function. For BAT metabolic activity study, BOLD MRI was able to measure BAT oxygenation effects [92,94,95], while hyperpolarized Xenon MRI [96,97], and contrast-enhanced MRI could measure BAT perfusion [131,132,133]. In this review, we focus on the methods that could assess BAT volume/mass but not function.

Chemical shift encoding-based water-fat MRI techniques allowed simultaneous mapping of proton density fat fraction (PDFF) and T2* for adipose tissue. BAT was reported with a lower PDFF and a shorter T2* compared with WAT [134,135,136,137]. Chemical shift MRI was widely employed to differentiate BAT from WAT; however, whether PDFF and T2* MRI could assess BAT volume independent of activation remains unclear. Since different biomarkers were used for assessing PDFF and T2* values, the situations were different as well.

**Figure 5:**
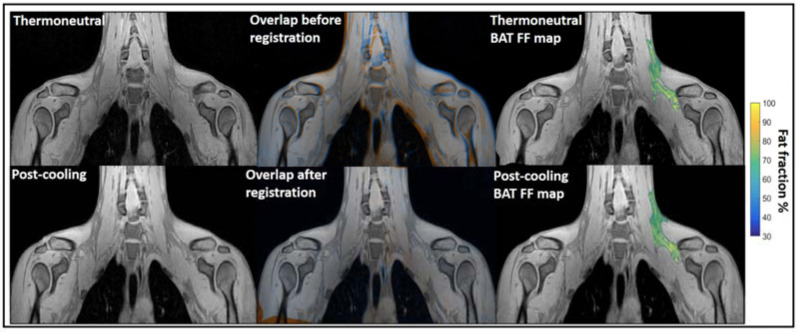
Example of image registration and a reconstructed fat fraction map before and after cooling. The first column shows thermoneutral and post-cooling images (one slice from the first echo in the acquisition). In the second column, the overlay of the same images before (top) and after registration (bottom) is shown. The images are colored orange (thermoneutral) and blue (post-cooling) for better visualization of differences between the scans. The third column shows the thermoneutral and post-cooling fat fraction maps of the supraclavicular adipose depot, overlaid on the corresponding images. Lipid content in the supraclavicular region is color-mapped over a 30–100% fat fraction range. Reprinted with permission from reference [85]. Copyright © 2021 Abreu-Vieira, Sardjoe Mishre, Burakiewicz, Janssen, Nahon, van der Eijk, Riem, Boon, Dzyubachyk, Webb, Rensen and Kan.

**Water–fat MRI.** Chemical-shift-encoded MRI which based on the quantification of water and fat composition in the tissue is the most frequently used MRI to assess BAT morphology [134,135,136,137]. It has been widely used for BAT detection in both rodents and humans. Osculati et al. had first defined BAT deposits in living rats in 1989 with water–fat MRI by means of T1 weighted-spin-echo pulses [71]. Then, they continued improving their methodology with chemical shift imaging techniques aiming to discriminate BAT from other tissues [72] and further quantify the fat and water content of BAT tissue in rodents [73]. Hu et al. found greater BAT PDFF in obese animals than lean animals with chemical shift MRI [74]. Besides, water–fat MRI was also widely used to assess BAT morphology in humans across all ages. Potkin et al. demonstrated the reliability of using multi-echo water–fat MRI in 22 human neonates with a mean age of around 24 days for quantification throughout the torso of BAT depot volume and fat fraction measurements [75]. Enerback et al. showed evidence for an anatomically distinguishable interscapular BAT depot in human infants for the first time by using a combination of water–fat MRI and histological and biochemical analyses [76]. Hu et al. reported the unique depiction of supraclavicular BAT by water–fat chemical shift MRI and CT in a human three-month-old infant [77]. Moreover, Hu et al. imaged BAT and WAT of 39 children with chemical-shift-encoded water–fat MRI and compared fat fractions (FFs) in two infants. Their results showed infants had lower supraclavicular FFs than children [78]. Chu et al. demonstrated the feasibility of using joint FFs and T2* intensity values to distinguish BAT from WAT in 24 adolescents [79]. For adult studies, chemical-shift-encoded water–fat MRI could not only assess BAT distribution and volume in a thermoneutral condition [80,81,82], but also could be used as a tool for monitoring lipid change under cold stimulation, which means the PDFF values varied after cold exposure [83,84,85,86,87,88,89]. Therefore, it is still controversial whether water–fat MRIs could reliably assess BAT volume independent of activation. Only a few reports were available in which FFs was not influenced by temperature. With PET-MRI imaging, Holstila et al. found cold exposure did not significantly affect MR-based measurement. Both BAT PDFF and T2* were not changed significantly after cold exposure [90]. Very interestingly, in a report by Welch et al. with PET-CT and MRI imaging, although there is significant change in BAT PDFF for a whole group with 17 subjects after cold exposure, neither the BAT PDFF change in 12 normal subjects nor five overweight subjects were significant [91]. Unlike rodents which have well-defined and confined BAT depots, the BAT in adult humans have great heterogeneity. Therefore, their response to cold exposure must be heterogeneous as well. Towse et al. reported that voxels with initial FFs values of 60%–100% exhibited a significant decrease in FFs while a simultaneous increase in FFs occurred in voxels with initial FFs values of 0%–30% after cold exposure [84]. Similarly, Kan et al. found no cold induced FFs change in the fat fraction range of 30%–100%, but significant change in 50%–100% and 70%–100% [85] (Figure 5). The above literature indicated that the heterogeneity of the BAT depot found in humans could be the main reason for the conflicting results. Due to the variability in response between lipid-rich and lipid poor regions, cares should be taken when applying fat fraction thresholds for MRI BAT analysis. 

T2* weighted MRI. Another potential MRI method to differentiate BAT and WAT is the measurement of T2* relaxation (T2*). The high abundance of iron-rich mitochondria and capillaries in BAT contribute to a faster de-phasing of transverse magnetization, resulting in lower T2* values compared with WAT. Meanwhile, T2* mapping has routinely been performed in combination with fat fraction mapping techniques that use a chemical shift encoding-based water–fat imaging method. Based on multiple reports, BAT showed shorter T2* and greater R2* (defined as the inverse of T2*) than WAT, both in mice and humans [74,78,79,80,82]. While several groups reported that T2* did not change significantly with acute cold exposure [85,86,89,90], others reported the use of T2* relaxation to measure BAT metabolic activity based on the changes of magnetic properties of hemoglobin with acute cold exposure [93,133]. Whether T2* weighted mapping could be employed to assess BAT mass without influence from cold exposure requires further investigation.

### 2.4. Near-Infrared Fluorescence Imaging (NIRFI)

In contrast to PET and MRI, near-infrared fluorescence imaging (NIRF) is nonradioactive, easy-to-use, and relatively inexpensive, and these advantages make it more suitable for large-scale preclinical research on living animals.

Since there is no definite target for BAT imaging, high throughput screening has become an effective method for the discovery of ligand and fluorescent dyes binding to BAT specifically. Kolonin et al. performed the screen with a combinatorial peptide library in mice, and characterized a peptide that can selectively bind to the vascular endothelium of BAT [98]. In vivo imaging with a fluorescent dye labeled peptide PEP3-IRDye800 showed the accumulation in BAT, and the signal was increased after cold treatment. Our group has also demonstrated that a top-down screening approach could be used for seeking near infrared fluorescence (NIRF) imaging probes for BAT [99]. We screened 38 NIRF dyes in total and found two curcumin analogues (CRANAD-2 and -3) that have relatively high uptakes in BAT. After further structural modification, CRANAD-29 was validated for its excellent capacity for BAT imaging. The in vivo data showed that CRANAD-29 could detect BAT activation under cold exposure. We further demonstrated that it was feasible to use spectral unmixing to differentiate the BAT mass and activation with CRANAD-29 [50]. Similarly, heptamethine indocyanine dye IR-786 was reported for BAT metabolic imaging under CL 316, 243 treatment [100].

**Figure 6:**
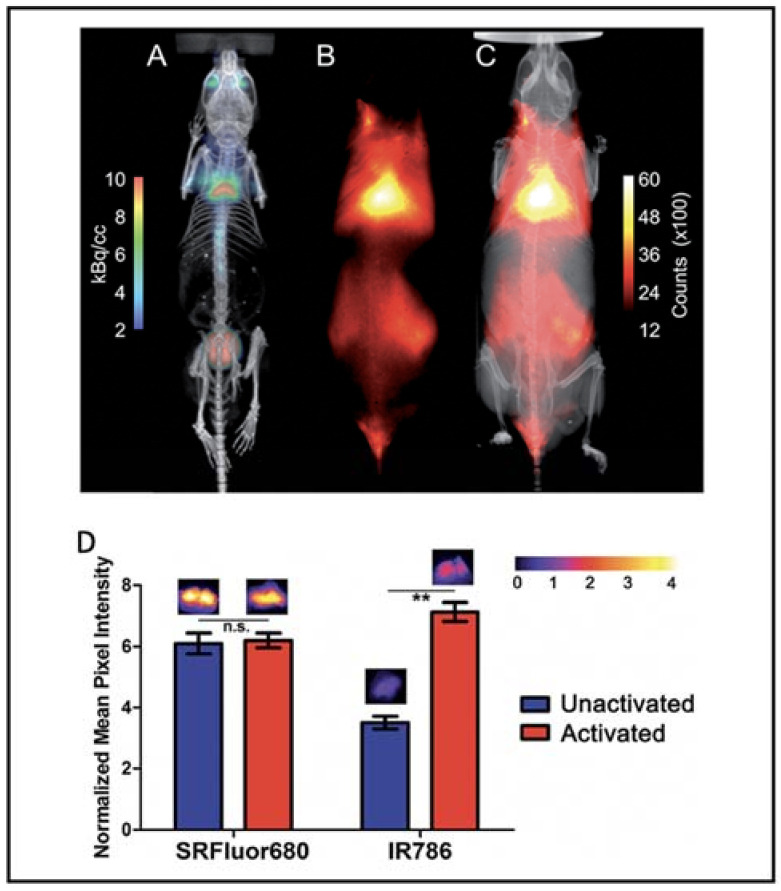
BAT imaging with micellar SRFluor680. (**A**) Representative PET/CT with ^18^F-FDG in a single living SKH1 mouse. (**B**,**C**) Fluorescence/X-ray images of the same SKH1 mouse with micellar SRFluor680. (**D**) Effect of BAT metabolic activation on probe accumulation. Representative fluorescence images of the excised BAT are shown above each of the bars. Note: n.s. (not significant), ** (*p* < 0.01). Reproduced from reference [103] with permission from the Royal Society of Chemistry.

Besides the small molecular dyes, several polymers were reported to be able to image BAT. Xiong et al. reported that fluorescent polymer dots MEH-PPV-NIR775 could image the whole-body BAT, including interscapular and axillar areas in living mice [101]. Moreover, polymer-modified single-walled carbon nanotube PMB-CNTs reported by Kataura et al. also could be used to image whole-body BAT [102]. However, whether their techniques are dependent on the metabolic state of BAT need to be determined. Smith et al. prepared micelle particles from SRFluor680, a commercially available deep-red fluorescent probe, to non-invasively image the interscapular BAT in mice [103]. Whole-body fluorescence imaging showed extensive accumulations of the fluorescent probe in the interscapular BAT, and ex vivo analysis showed 3.5-fold selectivity for interscapular BAT over interscapular WAT. Most importantly, additional imaging studies indicated that SRFluor680 uptake is independent of mouse species and the BAT metabolic state (Figure 6).

In addition, other optical approaches, including Cerenkov luminescence imaging, bioluminescence imaging, and photoacoustic imaging, were reported for BAT imaging. Our group has reported that Cerenkov luminescence imaging with ^18^F-FDG could be used to monitor BAT activation under different conditions [104,105]. Cheng et al. reported that CyHF-8 was capable of noninvasively detecting interscapular BAT with both NIRF and photoacoustic imaging [106]. Stahl et al. reported that the bioluminescence imaging probe FFA-SS-luc, a conjugate of long-chain fatty acids and firefly luciferin, could be used for bioluminescence BAT imaging, and they further demonstrated that the probe could be used to monitor fatty acid uptake under BAT activation conditions [107].

### 2.5. Contrast Enhanced Ultrasound (CEUS)

Contrast enhanced ultrasound (CEUS) is an attractive approach for the assessment of BAT due to its relatively low cost, no exposure to ionizing radiation, and the equipment is widely available in clinical settings. The advantages make it a potential imaging method for longitudinal studies involving pharmacologic interventions or natural history of disease. In 2013, Scherrer-Crosbie et al. demonstrated the feasibility of CEUS for the estimation of both BAT mass and BAT blood flow with norepinephrine stimulation [108]. In 2015, the same group reported the application of CEUS to assess BAT in healthy humans [109]. Their results showed an increased acoustic signal in the supraclavicular adipose tissue area that co-localized with BAT depot images of ^18^F-FDG PET-CT. In addition, the CEUS-derived BAT blood flow was increased with cold exposure compared to BAT blood flow at room temperature. Since the signal was dependent on stimulation, the CEUS could be a promising clinically relevant technique for BAT function study; however, this method may have limitations for BAT mass monitoring.

### 2.6. Other Imaging Modalities for BAT Imaging

Beside the traditional imaging modalities, several new imaging techniques emerged specifically for BAT imaging, and these methods could be used for studying BAT function. Two near-infrared spectroscopy (NIRS) techniques were commonly used for BAT properties monitoring: near-infrared time resolved spectroscopy (NIRTRS) that targets the oxygenated and deoxygenated hemoglobin and near-infrared continuous wavelength spectroscopy (NIRcws) that measures the relative oxygenation changes in the tissue [110]. The NIRS methods were used for estimating the changes of human BAT metabolic characteristics, including oxygenated hemoglobin, deoxygenated hemoglobin, total hemoglobin, hemoglobin oxygen saturation and a reduced scattering coefficient under different stimulations and after thermogenic food supplementation [111,112,113,114].

Infrared thermography (IRT) has been studied since 2012 for the assessment of BAT thermogenesis due to the heat-generating properties of BAT. The BAT thermogenesis under different stimulations including cold exposure, capsinoids injection, mental stress challenge and oral glucose tolerance test was monitored by IRT in separate studies [115,116,117,118,119,120,121,122,123,124,125]. The results indicate IRT could be a promising method for detecting BAT activation, not BAT mass.

## 3. Discussion and Future Perspectives

In recent years, the clinical interests in treatment of metabolic disorders through manipulating BAT are increasing. BAT has been reported as a potential target for management of not only obesity [5,16,24,39,138,139] but also diabetes [140,141,142,143], and more recently it has been reported to play important roles in non-alcoholic fatty liver disease (NAFLD) [144,145,146]. Molecular imaging methods that could assess BAT mass and activation will be powerful tools for assisting clinical studies. ^18^F-FDG-PET/CT is currently the most frequently used method for the detection and quantification of activated BAT in humans. However, different experimental protocols caused large variations in the imaging results, and it was difficult to directly compare results from different laboratories. Therefore, a brown adipose reporting criteria in imaging studies (BARCIST 1.0) was established to standardize the FDG-PET/CT BAT imaging. The limitations of FDG-PET/CT imaging highlight the importance of establishing valid and reproducible methods for detecting and quantifying BAT in humans. Besides FDG-PET/CT, most current approaches, such as MRI, SPECT, NIRFI, CEUS, NIRS and IRT, hold great promise for the assessment of BAT in preclinical and clinical studies. However, imaging methods that could detect BAT mass independent of activation are still in shortage. When the ability to detect BAT mass is reported, it is supposed that the detected mass signal will not be affected by stimulation conditions. However, currently, only few methods could be used for reporting absolute BAT mass. Although numerous imaging methods are able to visualize BAT under thermoneutral conditions, the imaging signal changes with different simulation conditions, suggesting it is complicated to use these methods to report BAT mass. It is likely that the imaging signal of these methods is a mixture of signals from blood flow in BAT and BAT itself. If we could dissect the contributions from the two components, these imaging methods hold great promise to report BAT mass and BAT function/activation. On the other hand, the imaging methods based on glucose metabolism, fatty acid uptake, blood flow, oxygen consumption, and temperature change could have a limited capacity for BAT mass imaging, because these targets are very easy to be influenced by environmental and hormonal conditions.

Stably expressed proteins or receptors in BAT could be good biomarkers for BAT mass imaging. TSPO, a translocator protein expressed in mitochondria, was demonstrated to be a reliable biomarker for BAT mass imaging in mice. A retrospective study in humans with TSPO PET tracer also indicated the possibility for BAT mass imaging; however, more studies for investigation of BAT mass imaging under stimulation conditions in humans are needed. A complete determination of the thermogenic potential of human BAT requires not only assessment of BAT following acute stimulation, but also BAT in its basal state. A single imaging method could have limitations to accurately report BAT mass and BAT activation/function at the same time, and a combination of different methods or modalities could be the trend for monitoring both the BAT mass and metabolic state change in future research.

## 4. Conclusions

In summary, BAT can be detected through different imaging modalities, and molecular imaging has been very useful for understanding BAT biology and function. However, lack of noninvasive in vivo methods for BAT mass quantification is still one obstacle in BAT-related studies. Further research should put more effort toward the development of new imaging modality targeting stable expressed biomarkers in BAT, and toward optimizing the current approaches to translate them in clinical applications.

## Figures and Tables

**Table 1:** Overview of imaging methods in BAT detection.

Imaging Modality	Imaging Mechanism	Imaging Subject	BAT Quantitative Imaging	BAT Mass Detection Independent of Metabolic State	Limitations	References
PET ^18^F-FDG	Glucose metabolism	Rodent/Human	Yes	No	Ionizing radiation, high cost, easily affected by imaging conditions	[4,5,13,14,51]
^18^F-THA	Fatty acid uptake	Rat/ Human	Yes	No	Ionizing radiation, BAT activation dependent	[52,53]
^18^F-F-DA	Dopamine analogue	Human	Yes	ND	Ionizing radiation, high cost, no report on BAT activation	[54]
^18^F-FBnTP	Mitochondria membrane potential	Rat	Yes	No	Ionizing radiation, high cost, low SNR	[55,56]
^18^F-FMPEP-d2	Cannabinoid receptor-1 ligand	Mouse	Yes	ND	Low SNR, high lung uptake, limited in study under cold temperature	[57]
^18^F-F-DPA	TSPO ligand	Mouse	Yes	Yes	Ionizing radiation, high cost	[58,59]
^18^F-F-FEPPA	TSPO ligand	Mouse	Yes	Yes	Ionizing radiation, no human studies	[60]
^18^F-F-PBR28	TSPO ligand	Mouse	Yes	No	Ionizing radiation, BAT activation depedent	[61]
^64^Cu-Dis	TSPO ligand	Mouse	Yes	Yes	Ionizing radiation, no human studies	[58]
^11^C-PBR28	TSPO ligand	Human	Yes	ND	Short half-life, limited in study under cold temperature	[62]
^11^C-acetate	Oxidative activity	Rat/ Human	Yes	No	Short half-life	[63]
^11^C-MRB	Norepinephrine transporter ligand	Rat/ Human	Yes	Yes	Short half-life	[64,65]
^15^O-O_2_	Oxygen consumption	Human	Yes	No	Short half-life, low SNR	[66]
SPECT ^99^mTc- tetrofosmin	Mitochondrial density	Human	Yes	No	Ionizing radiation, low resolution	[67]
^99^mTc-MIBI	Blood flow, Mitochondrial density	Mouse/ Human	Yes	ND	Ionizing radiation, low resolution	[68]
^123^I-or ^125^I-MIBG	Norepinephrine analogue sympathetic innervation	Rat/ Human	Yes	No	Ionizing radiation, low SNR	[69]
^123^I-or ^125^I-BMIPP	Fatty acid uptake	Mouse	Yes	No	Ionizing radiation, no human study	[70]
MRI Chemical shift MRI	Fat-water content	Rodent/Human	Yes	ND	Limited in BAT/WAT mixture differentiation	[71,72,73,74,75,76,77,78,79,80,81,82,83,84,85,86,87,88,89,90,91]
T_2_* mapping	Mitochondria and oxy-/deoxyhemoglobin	Mouse/ Human	Yes	ND	Limited in BAT/WAT mixture differentiation	[74,78,79,80,82,85,86,89,90,92,93]
BOLD	Oxygen consumption and blood flow	Rodent/Human	Yes	No	Susceptibility artifacts	[92,94,95]
Hyperpolarized Xenon MRI	Blood flow	Mouse	Yes	No	Limited in technique and availability	[96,97]
NIRF PEP3-IRDye80	Vascular endothelium	Mouse	Yes	No	Limited penetration depth, low SNR	[98]
CRANAD-29	CD36	Mouse	Yes	No	Limited penetration depth	[50,99]
IR-786	Vascular perfusion	Mouse	Yes	No	Low resolution	[100]
MEH-PPV-NIR775	Mitochondria	Mouse	Yes	No	Limited penetration depth	[101]
PMB-CNTs	Vascular endothelium	Mouse	Yes	ND	Low resolution	[102]
Micellar SRFluor680	Adipocytes	Mouse	Yes	Yes	Limited penetration depth, low SNR	[103]
Cerenkov imaging with ^18^F-FDG	Glucose metabolism	Mouse	Yes	No	Ionizing radiation, Low SNR	[104,105]
CyHF-8	Dense vascularized network	Mouse	Yes	ND	Limited penetration depth	[106]
FFA-SS-luc	Fatty acid uptake	Mouse	Yes	ND	Low resolution, low SNR	[107]
CEUS	Blood flow	Mouse/ Human	Yes	No	Limited penetration depth, low SNR	[108,109]
NIRS	Tissue perfusion	Human	Yes	No	Limited penetration depth, low SNR	[110,111,112,113,114]
IRT	Temperature	Human	Yes	No	Limited penetration depth	[115,116,117,118,119,120,121,122,123,124,125]

Note: PET, positron emission tomography; ^18^F-FDG, 2-deoxy-2-^18^F-fluoroglucose; ^18^F-THA, 14-(R,S)-^18^F-fluoro-6-thiaheptadecanoic acid; ^18^F-F-DA, ^18^F-6-fluorodopamine; ^18^F-FBnTP, ^18^F-fluorobenzyltriphenyl phosphonium, ^18^F-FMPEP-d2, ^18^F-(3R,5R)-5-(3-(18F-fluoromethoxy)phenyl)-3-(((R)-1-phenylethyl)amino)-1-(4-(trifluoromethyl) phenyl)pyrrolidin-2-one; ^18^F-F-DPA, *N*,*N*-diethyl-2-(2-(4-(18F-fluoro)phenyl)-5,7-dimethylpyrazolo[1,5-a]pyrimidin-3-yl)acetamide; ^64^Cu-Dis, ^64^Cu-Disulfiram; ^11^C-MRB, (S,S)-^11^C-O-methylreboxetine; SPECT, single-photon emission imaging computerized tomography; ^99^mTc-MIBI, ^99^mTc-methylisobutylisonitrile; ^123^I-MIBG, ^123^I-meta-iodobenzylguanidine; ^125^I-BMIPP, ^125^I-b-methyl-p-iodophenyl-pentadecanoic acid; MRI, magnetic resonance imaging; BOLD, blood-oxygen-level dependent; NIRFI, near infrared fluorescence imaging; CEUS, contrast enhanced ultrasound; NIRS, near infrared spectroscopy; IRT, infrared thermography; ND: not determined; SNR: signal to noise ratio.
